# Optimized *b*-value selection for the discrimination of prostate cancer grades, including the cribriform pattern, using diffusion weighted imaging

**DOI:** 10.1117/1.JMI.5.1.011004

**Published:** 2017-10-27

**Authors:** Sarah L. Hurrell, Sean D. McGarry, Amy Kaczmarowski, Kenneth A. Iczkowski, Kenneth Jacobsohn, Mark D. Hohenwalter, William A. Hall, William A. See, Anjishnu Banerjee, David K. Charles, Marja T. Nevalainen, Alexander C. Mackinnon, Peter S. LaViolette

**Affiliations:** a Medical College of Wisconsin, Department of Radiology, Milwaukee, Wisconsin, United States; b Medical College of Wisconsin, Department of Biophysics, Milwaukee, Wisconsin, United States; c Medical College of Wisconsin, Department of Pathology, Milwaukee, Wisconsin, United States; d Medical College of Wisconsin, Department of Urology, Milwaukee, Wisconsin, United States; e Medical College of Wisconsin, Department of Radiation Oncology, Milwaukee, Wisconsin, United States; f Medical College of Wisconsin, Department of Biostatistics, Milwaukee, Wisconsin, United States; g Medical College of Wisconsin, Department of Pharmacology and Toxicology, Milwaukee, Wisconsin, United States; h Medical College of Wisconsin, Department of Biomedical Engineering, Milwaukee, Wisconsin, United States

**Keywords:** apparent diffusion coefficient, prostate cancer, magnetic resonance imaging, diffusion-weighted imaging, Gleason

## Abstract

Multiparametric magnetic resonance imaging (MP-MRI), including diffusion-weighted imaging, is commonly used to diagnose prostate cancer. This radiology–pathology study correlates prostate cancer grade and morphology with common b-value combinations for calculating apparent diffusion coefficient (ADC). Thirty-nine patients undergoing radical prostatectomy were recruited for MP-MRI prior to surgery. Diffusion imaging was collected with seven b-values, and ADC was calculated. Excised prostates were sliced in the same orientation as the MRI using 3-D printed slicing jigs. Whole-mount slides were digitized and annotated by a pathologist. Annotated samples were aligned to the MRI, and ADC values were extracted from annotated peripheral zone (PZ) regions. A receiver operating characteristic (ROC) analysis was performed to determine accuracy of tissue type discrimination and optimal ADC b-value combination. ADC significantly discriminates Gleason (G) G4-5 cancer from G3 and other prostate tissue types. The optimal b-values for discriminating high from low-grade and noncancerous tissue in the PZ are 50 and 2000, followed closely by 100 to 2000 and 0 to 2000. Optimal ADC cut-offs are presented for dichotomized discrimination of tissue types according to each b-value combination. Selection of b-values affects the sensitivity and specificity of ADC for discrimination of prostate cancer.

## 1 Introduction

Prostate cancer is the second leading cause of cancer death in men in the U.S.^1^^,^^2^ and the third leading cause of cancer death worldwide.^3^ Treatment of prostate cancer is dependent on cancer severity. High-grade cancer is treated with radical prostatectomy, radiation treatment, or hormone therapy, whereas low-grade cancer may be monitored by active surveillance.^4^ Ideally, noninvasive clinical imaging should allow physicians to estimate cancer grade in advance of confirmatory biopsy of suspicious regions.

Pathologic grading of prostate cancer has changed in the past decade by a consensus of the International Society of Urological Pathology (ISUP). Cribriform glands were upgraded from Gleason pattern 3 to 4 (G3 and G4) due to their association with more aggressive cancer.^5^ Moreover, since 2001, 16 papers plus many abstracts have shown the cribriform pattern to be associated with more biochemical failure and adverse pathology, such as stage, margin status, and cancer volume, compared to noncribriform (small fused) acini.^6^

Cribriform pattern is most frequently found in the peripheral zone (PZ). Furthermore, it is commonly known that ∼75% of all types of prostate cancer occur in the PZ.^6^^,^^7^ The PZ appears differently from the transition zone on MRI and therefore, different acquisitions are optimal for different zones in the PI-RADS v2 scoring system. Diffusion-weighted imaging (DWI) is weighted more heavily in the PZ while T2 is more relevant to the transition zone.

Recent research has shown that different magnetic resonance imaging (MRI) sequences have been able to discriminate cancer grades. Apparent diffusion coefficient (ADC) images derived from DWI-MRI have been shown to discriminate between the Gleason grades.^8^^–^^10^ Additional MR sequences, such as T1, T2, MR spectroscopy, and dynamic contrast-enhanced imaging, add additional information for grade discrimination.^11^^–^^19^

This study investigates various b-value combinations used to calculate ADC to determine combinations that optimally discriminate cancer grades, including cribriform versus noncribriform type, in both the entire prostate and limited to the PZ. We use a radiologic–pathologic (Rad-Path) correlation to validate various ADC measures in 1369 pathologically classified regions from 210 whole-mount processed prostate slides from 39 patients.

## 2 Methods

### 2.1 Patient Population

Thirty-nine men undergoing radical prostatectomy for prostate cancer were recruited and provided informed consent for this prospective institutional review board (IRB) approved study. Patients ranged from 45 to 72 years of age (average 60). The average prostate specific antigen (PSA) measured prior to surgery was 8.2  ng/dl with a range from 2.8 to 27.5  ng/dl. Demographics and PSA scores are shown in Table 1. All patients underwent radical prostatectomy within 2 weeks of clinical imaging.

**Table 1:** Patient demographics. Race: African American (A), Caucasian (C), Hispanic (H).

Pt code	Age	PSA	Race	Gleason score
1	63	13.5	A	3+4
2	68	4.5	C	4+3
3	61	6.6	C	3+4
4	58	4.4	C	5+4
5	51	4.7	C	3+4
6	65	6.3	C	4+3
7	56	4.9	C	3+4
8	59	21.9	C	3+4
9	61	3	H	3+4
10	72	6.6	C	3+4
11	59	5.5	C	3+4
12	58	5	C	3+4
13	49	4.8	A	3+3
14	59	6.1	C	3+3
15	60	4.5	A	3+3
16	57	5.3	C	3+4
17	67	11	A	3+4
18	53	4.9	C	3+4
19	63	5.2	C	3+4
20	62	6.9	C	3+4
21	67	4.7	C	3+3
22	56	6.4	C	3+3
23	55	3.1	C	3+3
24	61	10.3	C	4+5
25	45	7.2	C	3+3
26	62	27.5	A	3+4
27	53	18.5	C	3+4
28	61	10.3	C	4+5
29	59	7.3	C	4+3
30	61	5	C	3+4
31	54	17.2	C	3+4
32	68	18.7	C	3+4
33	63	4.9	C	3+4
34	59	19	A	3+4
35	64	5.2	C	3+4
36	59	2.8	C	3+3
38	66	5.9	C	3+4
37	66	5	C	3+4
39	67	6.2	C	4+5
Average	60.2	8.2		

### 2.2 Clinical Imaging

All patients were scanned on the same 3T GE MR750 Signa MRI system (General Electric, Waukesha, Wisconsin) using an endorectal coil. Ten different b-values (0, 10, 25, 50, 80, 100, 200, 500, 1000, and 2000 with 3, 1, 1, 1, 2, 2, 4, 8, and 16 respective averages) were collected with field of view optimized and constrained undistorted single-shot DWI. Sixteen slices of 0.47×0.47×4  mm resolution were acquired during a 6 min 44 s acquisition for whole prostate coverage. For the purposes of simplifying the analysis and results interpretation, b-values of 0, 50, 100, 200, 500, 1000, and 2000 were chosen for further calculations, and ADC was calculated for each pair of b-values^20^ using Eq. (1). A monoexponential fit was also calculated using all seven b-values, implemented in MATLAB (Mathworks Inc. Natick, Massachusetts). The b=0 image was coregistered to the anatomical T2 image using FSL’s FLIRT command (FMRIB Toolbox, Oxford), followed by manual verification and adjustment, if necessary:

$$\mathrm{ADC}=\frac{-1}{b_{2}-b_{1}}\;\ln(\frac{S_{2}}{S_{1}}).$$ (1)

### 2.3 Tissue Preparation

**Fig. 1:**
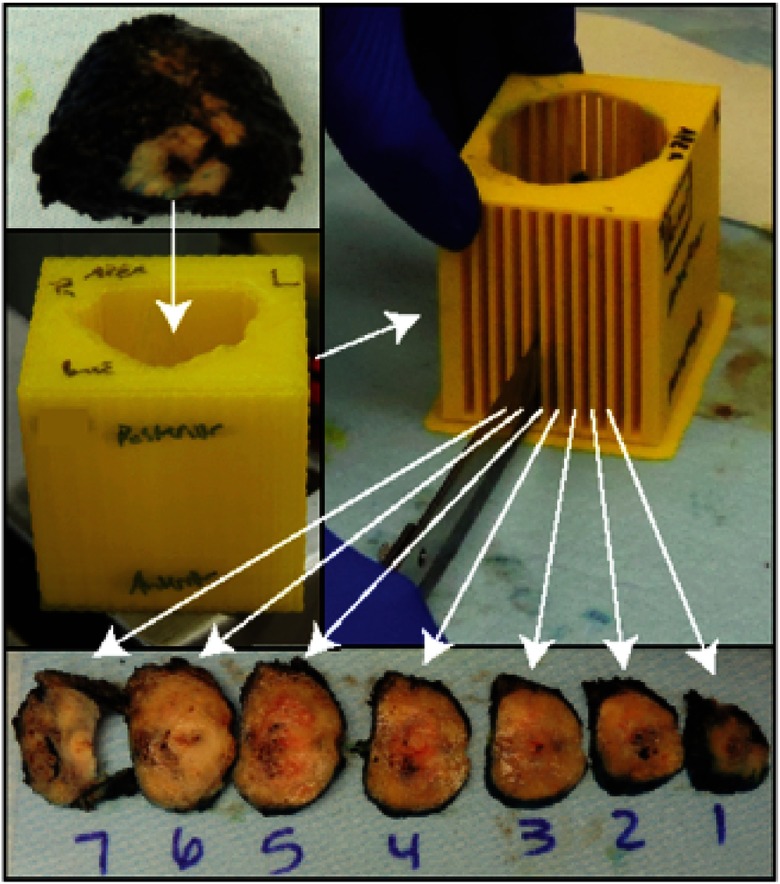
Demonstration of the use of patient-specific 3-D printed prostate slicing jig for sectioning tissue to match the orientation of anatomical presurgical MRI. The posterior surface of the prostate most distorted by the endorectal coil is oriented upward toward the mold insertion opening.

**Fig. 2:**
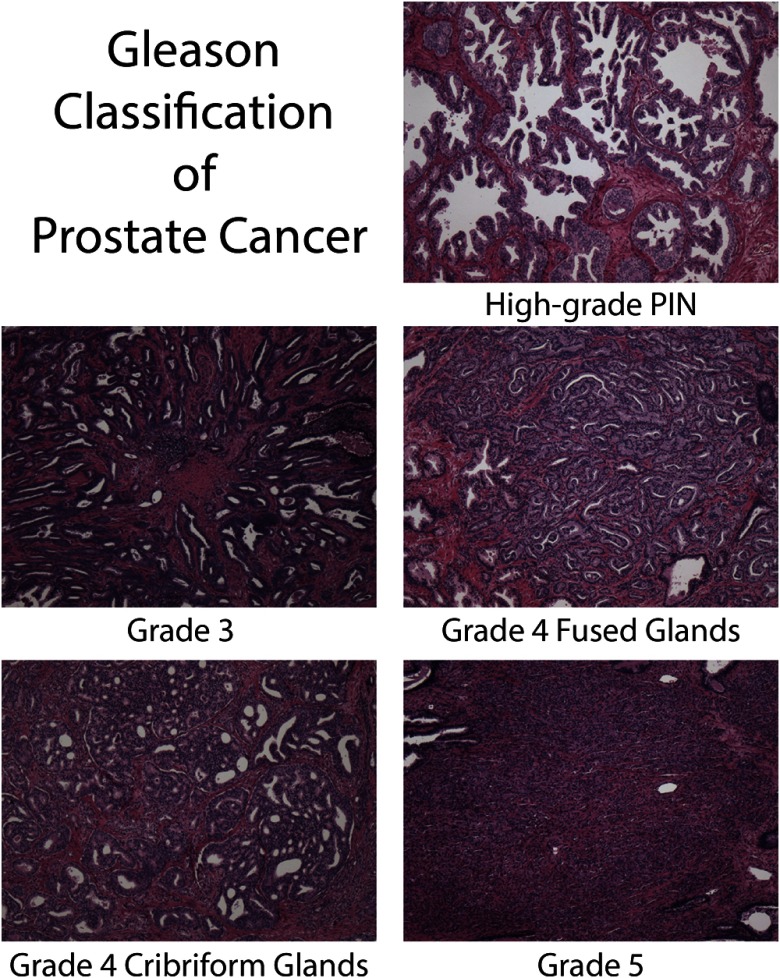
Examples of representative regions annotated using the Gleason system by our pathologist at 40× magnification.

**Fig. 3:**
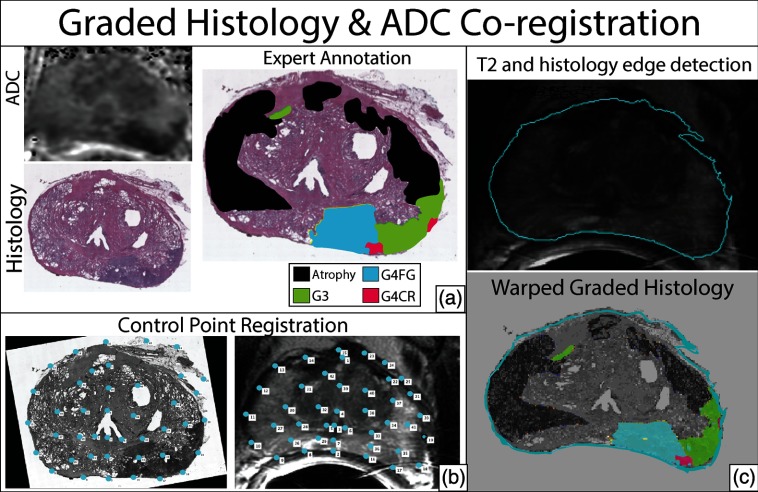
Demonstration of the (a) whole mount histology annotated by our urologic pathologist (b) coregistered to the T2 anatomic imaging using a control-point coregistration (c) with edge detection and applied warping to annotation and histology.

After radical prostatectomy, prostate samples were fixed in formalin overnight and then sectioned using patient-specific 3-D printed slicing jigs designed to match axial MRI orientation (Fig. 1).^21^ Each 4-mm tissue slice was paraffin-embedded in large format cassettes. A microtome was then used to slice 10  μm-thick sections, which were then transferred to whole-mount slides and hematoxylin and eosin stained. The slides were then digitized at 40× magnification using an automated microscope^22^ (Nikon, Tokyo, Japan). Digitized slides were annotated by a board-certified urologic pathologist using current modified Gleason grading.^5^^,^^23^^,^^24^ Regions of interest (ROIs) were manually drawn on the virtual slides creating annotated regions using different colors for: seminal vesicles, atrophy, high-grade prostatic intraepithelial neoplasia (HGPIN), G3, G4-fused small glands (G4FG), G4 cribriform glands (G4CG), and G5 carcinoma (Fig. 2). Regions not annotated were considered normal prostate. For subsequent analysis, normal prostate, seminal vesicles, atrophy, and HGPIN ROIs were combined and considered noncancerous (NC). Each digitized slide was then coregistered to the MRI and downsampled to corresponding voxel resolution (Fig. 3) using methods and software reported previously.^21^^,^^22^^,^^25^^,^^26^ Control point nonlinear coregistration was used to nonlinearly warp the histology and contoured annotation to match the endorectal coil compressed prostate in the T2-weighted MRI using the imwarp command in MATLAB (Mathworks Inc.).

### 2.4 Statistics

Mean and median ADC values for each b-value combination were extracted from ROIs defined by our pathologist. A receiver operating characteristic (ROC) analysis discriminating each cancer grade was then performed to determine the area under the curve (AUC) for each b-value combination. Two complimentary analyses were performed, one that included the entire prostate and one limited to the PZ. Comparisons included high-grade versus low-grade with noncancer (G4+ versus G3 and NC), high-grade versus low-grade (G4+ versus G3), G4CG versus NC, G4CG versus G3 and NC, and G4CG versus G3, limited to the PZ. Comparisons of annotated lesions within the entire prostate included G5 versus G3 cancer versus noncancer (G3+ versus NC), G4FG versus NCG5 versus NC, G4FG versus G3 and NC, G5 versus G3 and NC, and G4FG versus G3. The AUCs, cut-offs, p values, and mean and median values for each comparison group were generated for each b-value combination tested. An analysis of variance was also performed to determine the statistical significance comparing mean ADC values between grades on the entire dataset. Statistical tests were performed in SPSS (IBM Inc., Armank, New York) and ROC curves were generated using MATLAB (Mathworks Inc.).

## 3 Results

A total of 1369 annotated pathological regions from 210 prostate slices from 39 patients were included in the analysis of the entire prostate. This included 210 regions considered normal prostate tissue, 25 of seminal vesicles, 692 atrophies, 77 HGPIN, 252 G3, 81 G4FG, 24 G4CG, and 8 G5. When limited to the PZ, a total of 802 annotated pathological regions including 207 regions considered normal prostate tissue, 9 of seminal vesicles, 376 atrophies, 23 HGPIN, 127 G3, 38 G4FG, 16 G4CG, and 6 G5. Regions not annotated by our pathologist were considered normal.

**Fig. 4:**
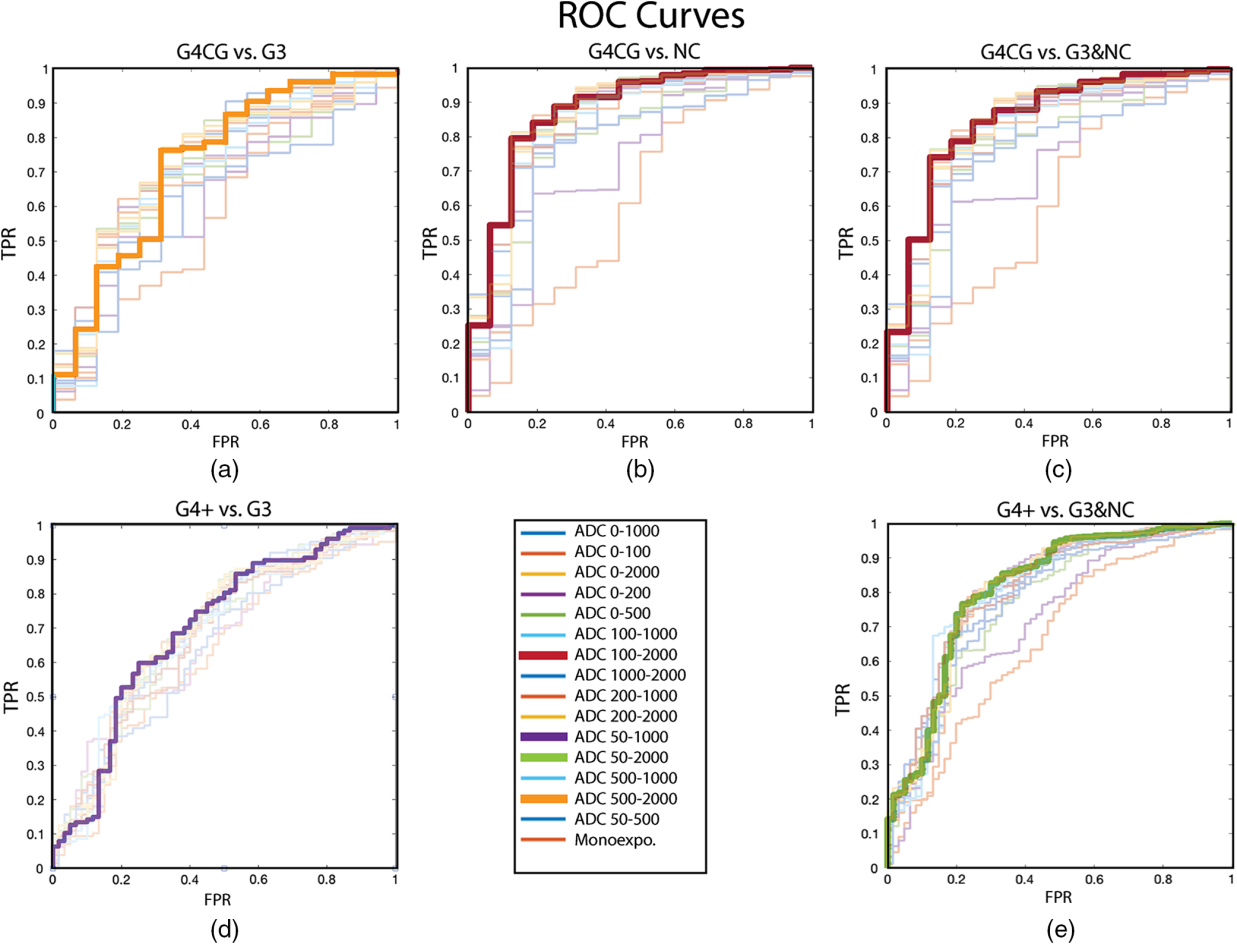
ROC curves plotted for each b-value combination against five different comparisons (HG versus G3 + noncancerous tissue, HG versus LG, cribriform glands versus NC tissue, cribriform glands versus LG, and cribriform glands versus G3 + NC tissue). The bold line represents the b-value combination with the highest AUC. AUC values are shown in Table 2. TPR, true-positive rate and FPR, false-positive rate. (a) G4CG versus G3, (b) G4CG versus NC, (c) G4CG versus G3 and NC, (d) G4+ versus G3, and (e) G4+ versus G3 and NC.

ADC alone significantly discriminates high-grade (G4+) cancer from low-grade (G3) and other normal tissue. The optimal b-value combinations and thresholds are shown in Table 2 (PZ) and Table 3 (whole prostate) marked by an asterisk. Figure 4 shows the ROC curves for G4+ versus G3 and NC, G4+ versus NC, G4CG versus NC, G4CG versus G3 and NC, and G4CG versus G3 within the PZ. Figure 6 shows the ROC curves for each combination performed in the whole prostate. Each b-value combination is plotted separately with the optimal combination highlighted by a thicker line.

**Table 2:** Analysis of the PZ. Summary table of ADC b-value combinations paired with the respective AUC, cut-off, and means and medians for each group being compared within the PZ of the prostate.

High grade (HG) versus low grade (LG) (G3)							
b-values	AUC	ADC cut-off	HG mean	LG mean	HG median	LG median	Asym Sig.
0–1000	0.701	1.273	1.306	1.443	1.263	1.442	0.000
0–100	0.634	2.218	2.066	2.323	1.956	2.229	0.003
0–2000	0.698	0.964	1.003	1.100	0.964	1.082	0.000
0–200	0.660	1.959	1.776	1.975	1.768	1.959	0.000
0–500	0.688	1.576	1.534	1.693	1.525	1.674	0.000
100–1000	0.689	1.163	1.221	1.345	1.196	1.345	0.000
100–2000	0.685	0.895	0.947	1.036	0.915	1.025	0.000
1000–2000	0.645	0.674	0.700	0.757	0.673	0.740	0.001
200–1000	0.693	1.131	1.188	1.310	1.146	1.314	0.000
200–2000	0.686	0.885	0.917	1.003	0.884	0.988	0.000
50–500	0.699	1.370	1.432	1.592	1.407	1.570	0.000
50–1000*	0.701	1.326	1.245	1.382	1.213	1.381	0.000
50–2000	0.696	0.934	0.966	1.062	0.929	1.052	0.000
500–1000	0.694	1.040	1.077	1.192	1.047	1.176	0.000
500–2000	0.678	0.808	0.826	0.902	0.794	0.884	0.000
Monoex	0.693	0.942	0.998	1.096	0.966	1.087	0.000
HG versus G3 and NC tissue							
b-values	AUC	ADC cut-off	HG mean	<G4 mean	HG median	<G4 median	Asym Sig.
0–1000	0.799	1.403	1.306	1.535	1.263	1.527	0.000
0–100	0.659	1.835	2.066	2.357	1.956	2.223	0.000
0–2000	0.812	1.060	1.003	1.176	0.964	1.170	0.000
0–200	0.722	1.953	1.776	2.064	1.768	2.015	0.000
0–500	0.767	1.600	1.534	1.779	1.525	1.767	0.000
100–1000	0.801	1.327	1.221	1.444	1.196	1.433	0.000
100–2000	0.807	1.017	0.947	1.114	0.915	1.107	0.000
1000–2000	0.780	0.781	0.700	0.816	0.673	0.818	0.000
200–1000	0.795	1.266	1.188	1.403	1.146	1.392	0.000
200–2000	0.801	0.975	0.917	1.077	0.884	1.071	0.000
50–500	0.781	1.578	1.432	1.691	1.407	1.670	0.000
50–1000	0.806	1.323	1.245	1.481	1.213	1.470	0.000
50–2000*	0.813	1.042	0.966	1.140	0.929	1.135	0.000
500–1000	0.804	1.205	1.077	1.291	1.047	1.279	0.000
500–2000	0.801	0.889	0.826	0.975	0.794	0.977	0.000
Monoex	0.810	1.066	0.998	1.175	0.966	1.167	0.000
G4 cribriform versus G3 and NC tissue							
b-values	AUC	ADC cut-off	G4CG mean	<G4 mean	G4CG median	<G4 median	Asym Sig.
0–1000	0.809	1.373	1.307	1.535	1.266	1.527	0.000
0–100	0.603	1.871	2.172	2.357	2.046	2.223	0.204
0–2000	0.842	0.990	0.986	1.176	0.960	1.170	0.000
0–200	0.707	1.927	1.792	2.064	1.778	2.015	0.008
0–500	0.768	1.591	1.545	1.779	1.544	1.767	0.001
100–1000	0.827	1.327	1.211	1.444	1.198	1.433	0.000
100–2000*	0.852	1.031	0.923	1.114	0.915	1.107	0.000
1000–2000	0.826	0.662	0.665	0.816	0.635	0.818	0.000
200–1000	0.812	1.235	1.186	1.403	1.183	1.392	0.000
200–2000	0.840	0.964	0.896	1.077	0.881	1.071	0.000
50–500	0.739	1.539	1.488	1.691	1.467	1.670	0.003
50–1000	0.800	1.323	1.268	1.481	1.238	1.470	0.000
50–2000	0.836	1.042	0.959	1.140	0.923	1.135	0.000
500–1000	0.809	1.159	1.069	1.291	1.049	1.279	0.000
500–2000	0.834	0.808	0.800	0.975	0.776	0.977	0.000
Monoex	0.842	1.084	0.984	1.175	0.964	1.167	0.000
G4 cribriform versus LG (G3)							
b-values	AUC	ADC cut-off	G4CG mean	LG mean	G4CG median	LG median	Asym Sig.
0–1000	0.704	1.286	1.307	1.443	1.266	1.442	0.057
0–100	0.582	1.872	2.172	2.323	2.046	2.229	0.579
0–2000	0.731	0.994	0.986	1.100	0.960	1.082	0.017
0–200	0.647	1.931	1.792	1.975	1.778	1.959	0.159
0–500	0.679	1.591	1.545	1.693	1.544	1.674	0.102
100–1000	0.705	1.331	1.211	1.345	1.198	1.345	0.050
100–2000*	0.736	0.953	0.923	1.036	0.915	1.025	0.012
1000–2000	0.719	0.662	0.665	0.757	0.635	0.740	0.007
200–1000	0.695	1.238	1.186	1.310	1.183	1.314	0.072
200–2000	0.728	0.934	0.896	1.003	0.881	0.988	0.018
50–500	0.630	1.542	1.488	1.592	1.467	1.570	0.403
50–1000	0.674	1.326	1.268	1.382	1.238	1.381	0.158
50–2000	0.714	0.929	0.959	1.062	0.923	1.052	0.037
500–1000	0.705	1.091	1.069	1.192	1.049	1.176	0.041
500–2000	0.728	0.808	0.800	0.902	0.776	0.884	0.010
Monoex	0.725	0.993	0.984	1.096	0.964	1.087	0.024
G4 cribriform versus NC tissue							
b-values	AUC	ADC cut-off	G4CG mean	NC mean	G4CG median	NC median	Asym Sig.
0–1000	0.830	1.373	1.307	1.554	1.266	1.540	0.000
0–100	0.607	1.871	2.172	2.364	2.046	2.219	0.172
0–2000	0.865	1.095	0.986	1.191	0.960	1.184	0.000
0–200	0.719	1.927	1.792	2.083	1.778	2.021	0.003
0–500	0.786	1.594	1.545	1.796	1.544	1.776	0.000
100–1000	0.852	1.327	1.211	1.464	1.198	1.445	0.000
100–2000*	0.876	1.031	0.923	1.130	0.915	1.122	0.000
1000–2000	0.848	0.774	0.665	0.829	0.635	0.824	0.000
200–1000	0.836	1.235	1.186	1.422	1.183	1.408	0.000
200–2000	0.863	0.966	0.896	1.092	0.881	1.086	0.000
50–500	0.761	1.539	1.488	1.711	1.467	1.687	0.001
50–1000	0.826	1.323	1.268	1.501	1.238	1.482	0.000
50–2000	0.861	1.042	0.959	1.156	0.923	1.148	0.000
500–1000	0.830	1.159	1.069	1.312	1.049	1.302	0.000
500–2000	0.856	0.810	0.800	0.990	0.776	0.988	0.000
Monoex	0.866	1.084	0.984	1.191	0.964	1.183	0.000

**Table 3:** PZ and TZ/CZ combined. Analysis of the entire prostate. Summary table of ADC *b*-value combinations paired with the respective AUC, cut-off, and means and medians for each group being compared over the entire prostate. HG = high grade, LG = low grade.

HG versus LG (G3)							
ADC	AUC	Cut-off	HG mean	LG mean	HG median	LG median	Asym. Sig.
0–1000	0.618	1.253	1.332	1.411	1.296	1.408	0.000
0–100	0.612	2.141	2.104	2.277	1.921	2.187	0.001
0–2000	0.609	0.965	1.016	1.072	0.987	1.065	0.001
0–200	0.607	1.578	1.812	1.945	1.772	1.910	0.001
0–500*	0.621	1.532	1.551	1.650	1.512	1.647	0.000
100–1000	0.606	1.193	1.245	1.313	1.215	1.302	0.001
100–2000	0.598	0.927	0.959	1.008	0.922	0.993	0.003
1000–2000	0.582	0.666	0.702	0.733	0.669	0.720	0.013
200–1000	0.609	1.143	1.209	1.277	1.173	1.253	0.001
200–2000	0.597	0.887	0.928	0.975	0.894	0.959	0.003
50–1000	0.623	1.233	1.271	1.351	1.230	1.341	0.000
50–2000	0.610	0.916	0.980	1.035	0.956	1.017	0.001
500–1000	0.607	1.163	1.108	1.169	1.067	1.147	0.001
500–2000	0.589	0.795	0.838	0.879	0.811	0.864	0.007
Monoex	0.604	0.946	1.000	1.053	0.979	1.039	0.001
G4-fused glands versus LG (G3)							
ADC	AUC	Cut-off	HG mean	LG mean	HG median	LG median	Asym. Sig.
0–1000	0.605	1.253	1.338	1.411	1.309	1.408	0.005
0–100	0.603	2.141	2.143	2.277	1.954	2.187	0.005
0–2000	0.590	0.965	1.024	1.072	1.014	1.065	0.015
0–200	0.589	1.578	1.842	1.945	1.799	1.910	0.016
0–500	0.616	1.425	1.553	1.650	1.513	1.647	0.002
100–1000	0.597	1.122	1.248	1.313	1.233	1.302	0.009
100–2000	0.581	0.885	0.966	1.008	0.949	0.993	0.029
1000–2000	0.564	0.666	0.712	0.733	0.669	0.720	0.085
200–1000	0.603	1.143	1.210	1.277	1.187	1.253	0.005
200–2000	0.583	0.882	0.934	0.975	0.905	0.959	0.025
50–1000*	0.617	1.165	1.272	1.351	1.265	1.341	0.002
50–2000	0.596	0.916	0.985	1.035	0.970	1.017	0.009
500–1000	0.587	1.163	1.119	1.169	1.105	1.147	0.019
500–2000	0.566	0.984	0.849	0.879	0.823	0.864	0.074
Monoex	0.587	0.946	1.007	1.053	0.986	1.039	0.018
G4 cribriform versus LG (G3)							
ADC	AUC	Cut-off	HG mean	LG mean	HG median	LG median	Asym. Sig.
0–1000	0.637	1.282	1.329	1.411	1.254	1.408	0.027
0–100	0.596	2.454	2.075	2.277	1.973	2.187	0.118
0–2000*	0.648	1.051	0.999	1.072	0.966	1.065	0.016
0–200	0.625	1.957	1.774	1.945	1.799	1.910	0.042
0–500	0.624	1.566	1.561	1.650	1.519	1.647	0.045
100–1000	0.625	1.262	1.242	1.313	1.187	1.302	0.043
100–2000	0.644	0.923	0.940	1.008	0.905	0.993	0.020
1000–2000	0.632	0.622	0.675	0.733	0.654	0.720	0.032
200–1000	0.624	1.237	1.210	1.277	1.174	1.253	0.045
200–2000	0.638	0.887	0.910	0.975	0.881	0.959	0.026
50–1000	0.625	1.230	1.282	1.351	1.215	1.341	0.042
50–2000	0.635	1.041	0.970	1.035	0.922	1.017	0.029
500–1000	0.641	1.065	1.090	1.169	1.043	1.147	0.022
500–2000	0.641	0.779	0.811	0.879	0.774	0.864	0.022
Monoex	0.644	1.037	0.983	1.053	0.949	1.039	0.019
G5 versus LG (G3)							
ADC	AUC	Cut-off	HG mean	LG mean	HG median	LG median	Asym. Sig.
0–1000	0.696	1.207	1.279	1.411	1.187	1.408	0.059
0–100*	0.750	2.055	1.793	2.277	1.796	2.187	0.016
0–2000	0.682	0.914	0.982	1.072	0.912	1.065	0.081
0–200	0.731	1.736	1.618	1.945	1.646	1.910	0.026
0–500	0.660	1.411	1.505	1.650	1.386	1.647	0.123
100–1000	0.644	1.144	1.226	1.313	1.134	1.302	0.165
100–2000	0.642	0.891	0.942	1.008	0.883	0.993	0.171
1000–2000	0.612	0.682	0.686	0.733	0.672	0.720	0.278
200–1000	0.627	1.072	1.198	1.277	1.122	1.253	0.222
200–2000	0.623	0.837	0.917	0.975	0.865	0.959	0.238
50–1000	0.684	1.158	1.234	1.351	1.149	1.341	0.076
50–2000	0.676	0.910	0.952	1.035	0.892	1.017	0.090
500–1000	0.707	1.043	1.051	1.169	1.014	1.147	0.046
500–2000	0.662	0.795	0.809	0.879	0.770	0.864	0.118
Monoex	0.657	0.919	0.975	1.053	0.910	1.039	0.130
Cancer versus NC tissue							
ADC	AUC	Cut-off	G5 mean	NC mean	G5 median	NC median	Asym. Sig.
0–1000	0.686	1.395	1.386	1.530	1.381	1.518	0.000
0–100	0.529	1.923	2.223	2.282	2.126	2.173	0.000
0–2000	0.708	1.044	1.055	1.174	1.041	1.166	0.000
0–200	0.590	1.698	1.904	2.026	1.861	1.981	0.000
0–500	0.656	1.550	1.619	1.769	1.616	1.756	0.000
100–1000	0.700	1.319	1.292	1.442	1.270	1.430	0.000
100–2000*	0.718	1.019	0.993	1.114	0.982	1.105	0.000
1000–2000	0.710	0.739	0.723	0.818	0.706	0.818	0.000
200–1000	0.700	1.271	1.256	1.401	1.233	1.390	0.000
200–2000	0.717	0.962	0.960	1.078	0.946	1.071	0.000
50–1000	0.696	1.321	1.326	1.477	1.308	1.464	0.000
50–2000	0.714	1.037	1.018	1.139	0.998	1.131	0.000
500–1000	0.697	1.176	1.150	1.289	1.125	1.282	0.000
500–2000	0.711	0.884	0.866	0.975	0.846	0.972	0.000
Monoex	0.714	1.040	1.037	1.158	1.025	1.151	0.000
G4-fused glands versus NC tissue							
ADC	AUC	Cut-off	G4FG mean	NC mean	G4FG median	NC median	Asym. Sig.
0–1000	0.739	1.400	1.338	1.530	1.309	1.518	0.000
0–100	0.606	1.921	2.143	2.282	1.954	2.173	0.001
0–2000	0.757	1.045	1.024	1.174	1.014	1.166	0.000
0–200	0.648	1.714	1.842	2.026	1.799	1.981	0.000
0–500	0.723	1.529	1.553	1.769	1.513	1.756	0.000
100–1000	0.746	1.341	1.248	1.442	1.233	1.430	0.000
100–2000	0.761	1.009	0.966	1.114	0.949	1.105	0.000
1000–2000	0.751	0.728	0.712	0.818	0.669	0.818	0.000
200–1000	0.749	1.223	1.210	1.401	1.187	1.390	0.000
200–2000	0.763	0.953	0.934	1.078	0.905	1.071	0.000
50–1000	0.756	1.310	1.272	1.477	1.265	1.464	0.000
50–2000*	0.767	1.009	0.985	1.139	0.970	1.131	0.000
500–1000	0.736	1.163	1.119	1.289	1.105	1.282	0.000
500–2000	0.752	0.871	0.849	0.975	0.823	0.972	0.000
Monoex	0.761	1.055	1.007	1.158	0.986	1.151	0.000
G4 cribriform versus NC tissue							
ADC	AUC	Cut-off	G4CG mean	NC mean	G4CG median	NC median	Asym. Sig.
0–1000	0.772	1.356	1.329	1.530	1.254	1.518	0.000
0–100	0.595	1.844	2.075	2.282	1.973	2.173	0.110
0–2000	0.810	1.049	0.999	1.174	0.966	1.166	0.000
0–200	0.691	1.657	1.774	2.026	1.799	1.981	0.001
0–500	0.743	1.564	1.561	1.769	1.519	1.756	0.000
100–1000	0.780	1.288	1.242	1.442	1.187	1.430	0.000
100–2000*	0.814	1.023	0.940	1.114	0.905	1.105	0.000
1000–2000	0.814	0.739	0.675	0.818	0.654	0.818	0.000
200–1000	0.773	1.236	1.210	1.401	1.174	1.390	0.000
200–2000	0.812	0.966	0.910	1.078	0.881	1.071	0.000
50–1000	0.768	1.374	1.282	1.477	1.215	1.464	0.000
50–2000	0.805	1.042	0.970	1.139	0.922	1.131	0.000
500–1000	0.765	1.087	1.090	1.289	1.043	1.282	0.000
500–2000	0.807	0.888	0.811	0.975	0.774	0.972	0.000
Monoex	0.810	1.036	0.983	1.158	0.949	1.151	0.000
Grade 5 versus NC tissue							
ADC	AUC	Cut-off	G5 mean	NC mean	G5 median	NC median	Asym. Sig.
0–1000	0.817	1.210	1.279	1.530	1.187	1.518	0.002
0–100	0.769	2.049	1.793	2.282	1.796	2.173	0.009
0–2000*	0.833	1.024	0.982	1.174	0.912	1.166	0.001
0–200	0.789	1.733	1.618	2.026	1.646	1.981	0.005
0–500	0.755	1.396	1.505	1.769	1.386	1.756	0.013
100–1000	0.790	1.142	1.226	1.442	1.134	1.430	0.005
100–2000	0.815	1.034	0.942	1.114	0.883	1.105	0.002
1000–2000	0.827	0.728	0.686	0.818	0.672	0.818	0.001
200–1000	0.758	1.172	1.198	1.401	1.122	1.390	0.012
200–2000	0.791	0.895	0.917	1.078	0.865	1.071	0.004
50–1000	0.808	1.159	1.234	1.477	1.149	1.464	0.003
50–2000	0.827	0.967	0.952	1.139	0.892	1.131	0.001
500–1000	0.828	1.044	1.051	1.289	1.014	1.282	0.001
500–2000	0.828	0.793	0.809	0.975	0.770	0.972	0.001
Monoex	0.820	1.077	0.975	1.158	0.910	1.151	0.002
HG versus G3 and NC tissue							
ADC	AUC	Cut-off	HG mean	<G4 mean	HG median	<G4 median	Asym. Sig.
0–1000	0.725	1.400	1.332	1.506	1.296	1.498	0.000
0–100	0.615	1.921	2.104	2.281	1.921	2.178	0.000
0–2000	0.741	1.045	1.016	1.154	0.987	1.147	0.000
0–200	0.655	1.714	1.812	2.010	1.772	1.969	0.000
0–500	0.708	1.564	1.551	1.745	1.512	1.733	0.000
100–1000	0.726	1.291	1.245	1.416	1.215	1.407	0.000
100–2000	0.740	1.009	0.959	1.093	0.922	1.084	0.000
1000–2000	0.732	0.735	0.702	0.801	0.669	0.802	0.000
200–1000	0.725	1.243	1.209	1.376	1.173	1.364	0.000
200–2000	0.740	0.966	0.928	1.057	0.894	1.053	0.000
50–1000	0.735	1.310	1.271	1.452	1.230	1.443	0.000
50–2000*	0.745	1.030	0.980	1.118	0.956	1.112	0.000
500–1000	0.720	1.163	1.108	1.265	1.067	1.253	0.000
500–2000	0.733	0.831	0.838	0.956	0.811	0.952	0.000
Monoex	0.741	1.040	1.000	1.137	0.979	1.127	0.000
G4-fused glands versus G3 and NC tissue							
ADC	AUC	Cut-off	HG mean	<G4 mean	HG median	<G4 median	Asym. Sig.
0–1000	0.712	1.400	1.338	1.506	1.309	1.498	0.000
0–100	0.606	1.921	2.143	2.281	1.954	2.178	0.001
0–2000	0.724	1.045	1.024	1.154	1.014	1.147	0.000
0–200	0.636	1.714	1.842	2.010	1.799	1.969	0.000
0–500	0.701	1.529	1.553	1.745	1.513	1.733	0.000
100–1000	0.716	1.341	1.248	1.416	1.233	1.407	0.000
100–2000	0.725	1.009	0.966	1.093	0.949	1.084	0.000
1000–2000	0.713	0.673	0.712	0.801	0.669	0.802	0.000
200–1000	0.720	1.223	1.210	1.376	1.187	1.364	0.000
200–2000	0.727	0.953	0.934	1.057	0.905	1.053	0.000
50–1000	0.728	1.310	1.272	1.452	1.265	1.443	0.000
50–2000*	0.733	1.009	0.985	1.118	0.970	1.112	0.000
500–1000	0.706	1.163	1.119	1.265	1.105	1.253	0.000
500–2000	0.714	0.831	0.849	0.956	0.823	0.952	0.000
Monoex	0.726	1.054	1.007	1.137	0.986	1.127	0.000
G4 cribriform versus G3 and NC tissue							
ADC	AUC	Cut-off	HG mean	<G4 mean	HG median	<G4 median	Asym. Sig.
0–1000	0.745	1.356	1.329	1.506	1.254	1.498	0.000
0–100	0.596	1.844	2.075	2.281	1.973	2.178	0.108
0–2000	0.778	1.049	0.999	1.154	0.966	1.147	0.000
0–200	0.678	1.956	1.774	2.010	1.799	1.969	0.003
0–500	0.719	1.564	1.561	1.745	1.519	1.733	0.000
100–1000	0.749	1.288	1.242	1.416	1.187	1.407	0.000
100–2000*	0.780	1.023	0.940	1.093	0.905	1.084	0.000
1000–2000	0.777	0.739	0.675	0.801	0.654	0.802	0.000
200–1000	0.743	1.236	1.210	1.376	1.174	1.364	0.000
200–2000	0.777	0.966	0.910	1.057	0.881	1.053	0.000
50–1000	0.740	1.374	1.282	1.452	1.215	1.443	0.000
50–2000	0.771	1.041	0.970	1.118	0.922	1.112	0.000
500–1000	0.740	1.086	1.090	1.265	1.043	1.253	0.000
500–2000	0.774	0.888	0.811	0.956	0.774	0.952	0.000
Monoex	0.777	1.036	0.983	1.137	0.949	1.127	0.000
G5 versus G3 and NC tissue							
ADC	AUC	Cut-off	HG mean	<G4 mean	HG median	<G4 median	Asym. Sig.
0–1000	0.793	1.207	1.279	1.506	1.187	1.498	0.004
0–100	0.765	2.049	1.793	2.281	1.796	2.178	0.010
0–2000	0.802	0.914	0.982	1.154	0.912	1.147	0.003
0–200	0.777	1.733	1.618	2.010	1.646	1.969	0.007
0–500	0.736	1.396	1.505	1.745	1.386	1.733	0.021
100–1000	0.761	1.142	1.226	1.416	1.134	1.407	0.011
100–2000	0.781	0.891	0.942	1.093	0.883	1.084	0.006
1000–2000	0.784	0.728	0.686	0.801	0.672	0.802	0.006
200–1000	0.732	1.172	1.198	1.376	1.122	1.364	0.024
200–2000	0.758	0.894	0.917	1.057	0.865	1.053	0.012
50–1000	0.783	1.158	1.234	1.452	1.149	1.443	0.006
50–2000	0.797	0.967	0.952	1.118	0.892	1.112	0.004
500–1000*	0.804	1.043	1.051	1.265	1.014	1.253	0.003
500–2000	0.795	0.793	0.809	0.956	0.770	0.952	0.004
Monoex	0.787	0.919	0.975	1.137	0.910	1.127	0.005

**Fig. 5:**
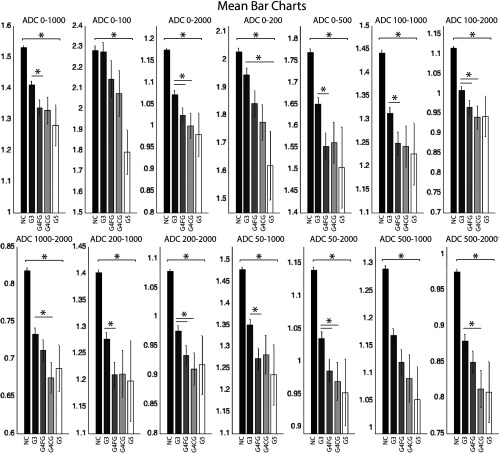
Bar charts indicating the mean ADC for each b-value combination within 1369 pathologist annotated regions including NC tissue, Gleason grade 3 (G3), grade 4 fused glands (G4FG), grade 4 cribriform (G4CG), and grade 5 (G5).

**Fig. 6:**
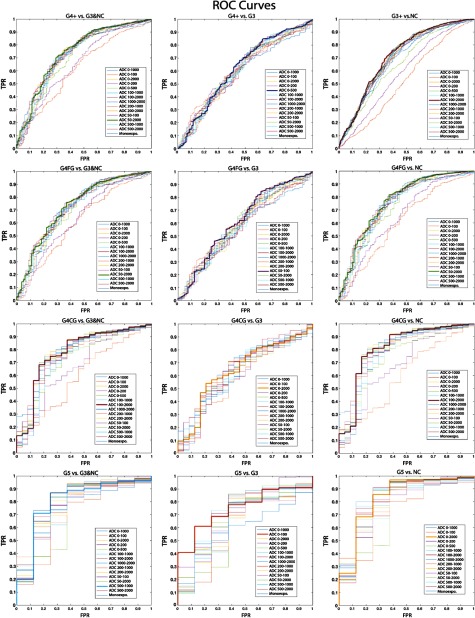
ROC curves plotted for each b-value combination against six different comparisons (HG versus G3 + NC tissue, HG versus LG, cancerous tissue (G3+) versus NC tissue, fused glands versus NC tissue, cribriform glands versus NC tissue, and grade 5 versus NC tissue). The bold line represents the b-value combination with the highest AUC. AUC values are shown in Table 3. (TPR, true-positive rate and FPR, false-positive rate.)

Results from the ROC analysis indicate ADC significantly discriminated high-grade cancer from low-grade and NC tissue. Figure 4 marks significant comparisons with an asterisk and Table 2 reports p values. Figure 5 shows that in general, diffusion is more restricted in higher grade cancer. However, comparison of G5 and G4CG or of G4CG and G4FG were not significant. Mean difference was significant between G5 and G3 using the b-value 0 to 200; G4CG and G3 using 0 to 2000, 100 to 2000, 1000 to 2000, 200 to 2000, 50 to 2000, and 500 to 2000; G4FG and G3 using 50 to 2000, 50 to 1000, 200 to 2000, 200 to 1000, 100 to 2000, 100 to 1000, 0 to 500, 0 to 2000, and 0 to 1000; and overall combinations between all high-grade cancers and NC tissue. The whole prostate analysis, including ROC curves from additional comparisons, is shown in Fig. 6 and Table 3, G5 versus G3 cancer versus noncancer (G3+ versus NC), G4FG versus NCG5 versus NC, G4FG versus G3 and NC, G5 versus G3 and NC, and G4FG versus G3.

The strongest b-value combination that was clinically relevant to discriminate high- from low-grade and NC tissue was 50 to 2000. For the splitting of individual Gleason grades, there was not a clear overall best combination, but inclusion of the highest b-value (e.g., 1000 to 2000, 50 to 2000, 0 to 2000) had larger AUCs. Specific values for each grade are shown in Table 2 for the PZ and Table 3 for the whole prostate, along with optimal cut-offs for all b-value combinations.

## 4 Discussion

This study used Rad-Path correlation to assess the sensitivity and specificity of various b-value combinations for calculating ADC to discriminate aggressive high-grade prostate cancer from indolent cancer. We found that in the PZ, the optimal b-value combination to discriminate high- from low-grade cancer and NC tissue was 50 to 2000, which resulted in an AUC of 0.813 with an optimal ADC threshold of $1.042\times 10^{-3}\;{\mathrm{mm}}^{2}/s$. Commonly acquired b-value combinations, such as 0 to 1000 and 0 to 2000, performed only marginally worse with AUC’s of 0.799 and 0.812, respectively.

Previous studies have shown that ADC is inversely correlated with cancer grade.^8^^,^^11^^,^^27^^,^^28^ These previous reports have grouped G4CG, G4FG, and G5 into one class of high-grade. This study divided these two grades into three subgroups to investigate the different diffusion properties of these three subgroups. Considering that cribriform pattern is more aggressive than G4FG, it was important to investigate our ability to identify it on MRI.

We found that mean ADC values for G4FG, G4CG, and G5 are not significantly different from one another although all three were significantly different from low-grade cancer in the higher performing b-value combinations (Fig. 5). Other studies have shown the same difficulty in separating G4 from G5, and therefore, merge G4 and G5 in their analysis.^8^^,^^11^ We found ADC alone can be used to discriminate each of these high-grade cancer types from all other prostate tissue types using b-value combinations. Importantly, cribriform glands hold a greater predictive value for biochemical failure than small fused glands.^29^ Our data show that lower ADC scores correlate with more severe cancer, with the exceptions reported above including G4CG not showing this perfect inverse correlation. The combinations most responsible for a variation from this inverse correlation are 0 to 500 and 1000 to 2000, where both group means and medians are altered, the former raising G4 cribriform, and the latter raising G5. This may be attributed to the diffusion compartments highlighted by these b-value combinations. More research is necessary to determine why the trend reverses.

We found that the inclusion of higher b-values in the ADC calculation resulted in increased sensitivity and specificity for differentiating high-grade cancer, which is supported by other work.^20^^,^^28^ The pathology of G5 is very different from the pathology of G4CG.^23^^,^^24^^,^^30^ G5 is characterized by infiltrating single cells, whereas G4CG has distended gland spaces with multiple lumens. A possible explanation for the variance in diffusion between these morphologies is that water may be able to diffuse through the stroma surrounding single cells more easily than through the highly cellular, thick-walled epithelium of cribriform glands.

There are several sources of potential error in our analysis. We chose to utilize an open design for the 3-D printed slicing jigs for ease of use. We initially experimented with a design similar to Trivedi et al.^31^ but found our pathology assistants preferred the design shown in Fig. 1 due to ease of use and minimal workflow disruption. Prostate samples fit within the mold snugly as the 3-D renderings used for the design were deflated subtly. It is plausible that small slice orientation discrepancies occurred. Future studies should intentionally perturb the coregistration scheme to determine downstream cancer classification effects. It will also be beneficial to the field to explore a more sophisticated fitting of the diffusion signal, and how the resulting maps effect cancer discrimination. An additional analysis of the intra- and intersubject variability of tumor ADC values would be an interesting direction for future research.

This study disclosed that while b-value selection to discriminate high-grade prostate cancer can be optimized, most common combinations perform suitably. ADC alone can be used to discriminate individual high-grade cancer types from low-grade cancer and NC tissue to a significant degree, with specific combinations outperforming others. We expect this information will guide clinical interpretation of ADC maps and spur additional research.

## References

[1] Lambin P. et al., “Radiomics: extracting more information from medical images using advanced feature analysis,” Eur. J. Cancer 48(4), 441–446 (2012). 10.1016/j.ejca.2011.11.036 22257792 PMC4533986

[2] Siegel R. L. Miller K. D. Jemal A., “Cancer statistics, 2016,” CA: Cancer J. Clin. 66(1), 7–30 (2016). CAMCAM 0007-9235 10.3322/caac.21332 26742998

[3] Lippi G. et al., “Prostate-specific antigen-based screening for prostate cancer in the third millenium: useful or hype?” Ann. Med. 41(7), 480–489 (2009). ANMDEU 0785-3890 10.1080/07853890903156468 19657768

[4] Bill-Axelson A. et al., “Radical prostatectomy versus watchful waiting in early prostate cancer,” N. Engl. J. Med. 364(18), 1708–1717 (2011). 10.1056/NEJMoa1011967 21542742

[5] Epstein J. I. et al., “The 2014 International Society of Urological Pathology (ISUP) consensus conference on Gleason grading of prostatic carcinoma: definition of grading patterns and proposal for a new grading system,” Am. J. Surg. Pathol. 40(2), 244–252 (2016). 10.1097/PAS.0000000000000530 26492179

[6] Weinreb J. C. et al., “PI-RADS prostate imaging: reporting and data system: 2015, version 2,” Eur. Urol. 69(1), 16–40 (2016). 10.1016/j.eururo.2015.08.052 26427566 PMC6467207

[7] Ravery V. et al., “Extensive biopsy protocol improves the detection rate of prostate cancer,” J. Urol. 164(2), 393–396 (2000). 10.1016/S0022-5347(05)67368-5 10893593

[8] Hoeks C. M. et al., “Prostate cancer: multiparametric MR imaging for detection, localization, and staging,” Radiology 261(1), 46–66 (2011). RADLAX 0033-8419 10.1148/radiol.11091822 21931141

[r9] Woodfield C. A. et al., “Diffusion-weighted MRI of peripheral zone prostate cancer: comparison of tumor apparent diffusion coefficient with Gleason score and percentage of tumor on core biopsy,” Am. J. Roentgenol. 194(4), W316–W322 (2010). 10.2214/AJR.09.2651 20308476

[10] Hosseinzadeh K. Schwarz S. D., “Endorectal diffusion-weighted imaging in prostate cancer to differentiate malignant and benign peripheral zone tissue,” J. Magn. Reson. Imaging 20(4), 654–661 (2004). 10.1002/(ISSN)1522-2586 15390142

[11] Hotker A. M. et al., “Assessment of prostate cancer aggressiveness by use of the combination of quantitative DWI and dynamic contrast-enhanced MRI,” Am. J. Roentgenol. 206, 756–763 (2016). 10.2214/AJR.15.14912 26900904 PMC5479568

[r12] Mazaheri Y. et al., “Prostate cancer: identification with combined diffusion-weighted MR imaging and 3D 1H MR spectroscopic imaging—correlation with pathologic findings,” Radiology 246(2), 480–488 (2008). RADLAX 0033-8419 10.1148/radiol.2462070368 18227542

[r13] Wang L. et al., “Assessment of biologic aggressiveness of prostate cancer: correlation of MR signal intensity with Gleason grade after radical prostatectomy,” Radiology 246(1), 168–176 (2008). RADLAX 0033-8419 10.1148/radiol.2461070057 18024440

[r14] Wu L. M. et al., “T2* relaxation time in the detection and assessment of aggressiveness of peripheral zone cancer in comparison with diffusion-weighted imaging,” Clin. Radiol. 71(4), 356–362 (2016). CLRAAG 0009-9260 10.1016/j.crad.2015.12.012 26823021

[r15] Metzger G. J. et al., “Detection of prostate cancer: quantitative multiparametric mr imaging models developed using registered correlative histopathology,” Radiology 279(3), 805–816 (2016). RADLAX 0033-8419 10.1148/radiol.2015151089 26761720 PMC4868764

[r16] Osugi K. et al., “What is the most effective tool for detecting prostate cancer using a standard MR scanner?” Magn. Reson. Med. Sci. 12(4), 271–280 (2013). 10.2463/mrms.2012-0054 24172787

[r17] Barentsz J. O. et al., “ESUR prostate MR guidelines 2012,” Eur. Radiol. 22(4), 746–757 (2012). EURAE3 1432-1084 10.1007/s00330-011-2377-y 22322308 PMC3297750

[r18] Oto A. et al., “Diffusion-weighted and dynamic contrast-enhanced MRI of prostate cancer: correlation of quantitative MR parameters with Gleason score and tumor angiogenesis,” Am. J. Roentgenol. 197(6), 1382–1390 (2011). 10.2214/AJR.11.6861 22109293

[19] Kozlowski P. et al., “Combined diffusion-weighted and dynamic contrast-enhanced MRI for prostate cancer diagnosis—correlation with biopsy and histopathology,” J. Magn. Reson. Imaging 24(1), 108–113 (2006). 10.1002/(ISSN)1522-2586 16767709

[20] de Perrot T. et al., “Diffusion in prostate cancer detection on a 3T scanner: how many b-values are needed?” J. Magn. Reson. Imaging 44(3), 601–609 (2016). 10.1002/jmri.v44.3 26914964

[21] Nguyen H. S. et al., “Progressing bevacizumab-induced diffusion restriction is associated with coagulative necrosis surrounded by viable tumor and decreased overall survival in patients with recurrent glioblastoma,” Am. J. Neuroradiol. 37(12), 2201–2208 (2016). 10.3174/ajnr.A4898 27492073 PMC5161572

[22] LaViolette P. S. et al., “Precise ex vivo histological validation of heightened cellularity and diffusion-restricted necrosis in regions of dark apparent diffusion coefficient in 7 cases of high-grade glioma,” Neuro-Oncology 16(12), 1599–1606 (2014). 10.1093/neuonc/nou142 25059209 PMC4232087

[23] Gleason D. F., “Classification of prostatic carcinomas,” Cancer Chemother. Rep. 50(3), 125–128 (1966). 5948714

[24] Iczkowski K. A. Lucia M. S., “Current perspectives on Gleason grading of prostate cancer,” Curr. Urol. Rep. 12(3), 216–222 (2011). 10.1007/s11934-011-0181-5 21424766

[25] McGarry S. et al., “Magnetic resonance imaging-based radiomic profiles predict patient prognosis in newly diagnosed glioblastoma before therapy,” Tomography 2(3), 223 (2016). 10.18383/j.tom.2016.00250 27774518 PMC5074084

[26] Nguyen H. S. et al., “Progressing bevacizumab-induced diffusion restriction is associated with coagulative necrosis surrounded by viable tumor and decreased overall survival in patients with recurrent glioblastoma,” Am. J. Neuroradiol. 37(12), 2201–2208 (2016). 10.3174/ajnr.A4898 27492073 PMC5161572

[27] Osugi K. et al., “What is the most effective tool for detecting prostate cancer using a standard MR scanner?” Magn. Reson. Med. Sci. 12(4), 271–280 (2013). 10.2463/mrms.2012-0054 24172787

[28] Peng Y. et al., “Apparent diffusion coefficient for prostate cancer imaging: impact of B values,” Am. J. Roentgenol. 202(3), W247–W253 (2014). 10.2214/AJR.13.10917 24555621

[29] Siadat F. et al., “Not all Gleason pattern 4 prostate cancers are created equal: a study of latent prostatic carcinomas in a cystoprostatectomy and autopsy series,” Prostate 75(12), 1277–1284 (2015). PRSTDS 1097-0045 10.1002/pros.v75.12 25963383

[30] Epstein J. I. et al., “A contemporary prostate cancer grading system: a validated alternative to the Gleason score,” Eur. Urol. 69(3), 428–435 (2015). 10.1016/j.eururo.2015.06.046 26166626 PMC5002992

[31] Trivedi H. et al., “Use of patient-specific MRI-based prostate mold for validation of multiparametric MRI in localization of prostate cancer,” Urology 79(1), 233–239 (2012). 10.1016/j.urology.2011.10.002 22202553 PMC3534884

